# Enhancing Fucoxanthin Pickering Emulsion Stability and Encapsulation with Seaweed Cellulose Nanofibrils Using High-Pressure Homogenization

**DOI:** 10.3390/md23080311

**Published:** 2025-07-30

**Authors:** Ying Tuo, Mingrui Wang, Yiwei Yu, Yixiao Li, Xingyuan Hu, Long Wu, Zongpei Zhang, Hui Zhou, Xiang Li

**Affiliations:** 1 College of Food Science and Engineering, Dalian Ocean University, Dalian 116023, China; 2 Dalian Jinshiwan Laboratory, Dalian 116034, China; 3 National R&D Branch Center for Seaweed Processing, Dalian Ocean University, Dalian 116023, China; 4 Qingdao Bright Moon Seaweed Group Co., Ltd., Qingdao 266499, China

**Keywords:** fucoxanthin, cellulose nanofibrils, Pickering emulsion, encapsulation, stability

## Abstract

Poor solubility and bioavailability have limited the application of fucoxanthin in drug and functional food processing. In order to encapsulate fucoxanthin in delivery systems, in this study, cellulose was isolated from industrial brown algae residues and high-pressure homogenized into cellulose nanofibrils (CNFs). Then, fucoxanthin was encapsulated into the Pickering emulsion stabilized by the CNFs. The effect of high-pressure homogenization on the characteristics of cellulose and the stability of fucoxanthin emulsion was evaluated. The results indicated that CNFs prepared at 105 MPa had a diameter of 87 nm and exhibited high zeta potential and thermal stability. Encapsulation efficiency peaked at 70.8% with 1.0 mg/mL fucoxanthin, and after three freeze–thaw cycles the encapsulation efficiency was higher than 60%. The DPPH scavenging activity after 12 days’ storage at 4 °C was still 42%. Furthermore, the Pickering emulsion with 1.0 mg/mL fucoxanthin showed high stability and antioxidant activity under different pH values, salinity, temperature, and UV light exposure duration. The CNFs effectively protected fucoxanthin from degradation, offering a novel delivery system for marine bioactive compounds. To the best of our knowledge, this is the first study on the fucoxanthin delivery system of Pickering emulsion stabilized by the CNFs. Such emulsion might benefit the encapsulation and release of bioactive components in marine drugs.

## 1. Introduction

Fucoxanthin is a kind of natural carotenoid extracted from brown algae and has a series of bioactive functions, such as antioxidant and antitumor activity [1]. The bioactive functions of fucoxanthin are related to its unique structure, which is composed of an allelic bond, a 5,6- monoepoxide, and conjugated double bonds [2]. However, the unique structure also leads to instability during processing and storage [3]. The poor bioavailability and instability of fucoxanthin in the gastrointestinal tract are major limitations [4]. In order to achieve the encapsulation protection, dispersion utilization, and delivery release of fucoxanthin in food and drug systems, a new delivery system for enhanced bioactive compound delivery in vivo needs to be developed.

Emulsions stabilized by solid particles instead of surfactants are known as Pickering emulsions [5]. Compared with conventional surfactant emulsions, the Pickering emulsions systems display superior storage stability [6]. Currently, natural biopolymers such as polysaccharides [7], proteins [8], and cellulose [9] have been proven to be capable of stabilizing Pickering emulsions. Moreover, emulsion delivery systems could effectively encapsulate fucoxanthin and enhance the bioavailability of fucoxanthin [10]. Furthermore, the interfacial film might also protect fucoxanthin from oxidation during storage [11]. Current reports on fucoxanthin emulsions mainly focus on preparation optimization [12] and in vitro digestion simulation [13]. However, the long-term storage stability of fucoxanthin in emulsion has not been fully evaluated.

Cellulose is the most abundant renewable natural green material on Earth, with advantages such as high thermal stability and biodegradability [14]. Nanocellulose is produced from natural cellulose by physical or chemical treatments, and it features at least one dimension in the 1–1000 nm range [15]. Nanocellulose exhibits a high aspect ratio and strong interfacial adsorption. The three-dimensional network structure facilitates nanocellulose efficient loading and controlled release of bioactive compounds. Recent studies have demonstrated the potential of natural biopolymers, including cellulose nanofibrils, to stabilize Pickering emulsions. For instance, Burgos-Diaz et al. reported the use of starch-based nanoparticles to stabilize Pickering emulsions for astaxanthin encapsulation, showing improved stability and bioaccessibility [16]. Similarly, Gan et al. demonstrated that TEMPO-oxidized cellulose nanofibrils combined with chitosan can significantly enhance the stability and bioavailability of astaxanthin through the formation of pH-responsive nanoparticles [17]. These studies suggest cellulose can effectively encapsulate bioactive compounds, enhancing stability and bioavailability. Seaweed cellulose, isolated from industrial waste, is a high-cellulose-content byproduct with potential as a stabilizer. This study investigates the impact of high-pressure homogenization on industrial brown algae residues for stabilizing fucoxanthin Pickering emulsions.

Currently, reports on fucoxanthin emulsions mainly focus on preparation optimization and in vitro digestion simulation. However, the long-term storage stability of fucoxanthin in emulsions has not been fully explored. Moreover, the existing delivery systems for fucoxanthin often suffer from limitations such as poor encapsulation efficiency and insufficient protection against environmental factors during processing and storage. To address these issues, this study hypothesizes that high-pressure homogenization of seaweed cellulose into cellulose nanofibrils (CNFs) can enhance the stability and encapsulation efficiency of fucoxanthin in Pickering emulsions. The expected outcomes include improved stability of fucoxanthin under various processing conditions, enhanced encapsulation efficiency, and better retention of bioactivity. This study aims to develop a novel and effective delivery system for fucoxanthin using seaweed CNFs. This new system is designed to overcome the limitations of existing systems and provide a stable and efficient encapsulation platform for bioactive compounds in marine drugs and functional foods. In the future, cytotoxicity and biocompatibility assessments will be performed or planned to support pharmaceutical applications in the next study. To the best of our knowledge, this is the first study on the fucoxanthin delivery system of Pickering emulsion stabilized by CNFs. This research advances sustainable marine delivery platforms for nutraceuticals and fortified foods, bridging the gap between laboratory innovation and practical application.

## 2. Results and Discussion

### 2.1. Effect of Homogenization Pressure on Polymerization Degree of Brown Seaweed Cellulose

The polymerization degree denotes the number of glucose units that were connected in the molecular chain of cellulose. The polymerization degree reflects on the length of the chain of cellulose and significantly affects the physicochemical characteristics of the CNFs. As shown in Table 1, the homogenization pressure significantly affects the polymerization degree of CNFs. The polymerization degree of CNFs without high-pressure homogenization (H-0) was 331.24 ± 3.6, which is significatively (*p* < 0.05) higher than that of microcrystalline cellulose (MCC, 170.82 ± 4.1). The polymerization degree of CNFs in the present study was lower than that of cellulose of *Kjellmaniella crassifolia* (444.60) [18]. With the increasing pressure of homogenization from 35 to 140 MPa, the low polymerization degree of CNFs could be observed in the results. The CNFs with 140 MPa homogenization (H-140) showed the lowest polymerization degree of 264.94 ± 18.5. The high shear and impact forces of high-pressure homogenization might contribute to such results, cutting the carbohydrate chain of cellulose into shorter CNFs [19]. The results of the polymerization degree might suggest the effect on the particle size of CNFs and attribute it to the interface stability in Pickering emulsion.

### 2.2. Effect of Homogenization Pressure on Crystalline Structure of Brown Seaweed Cellulose

**Figure 1:**
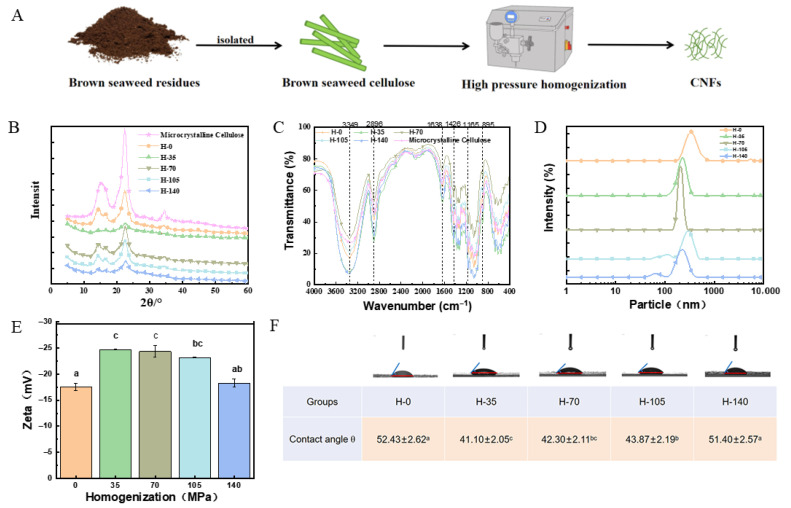
Preparation and characterization of CNFs derived from brown seaweed residues under various homogenization pressures. (**A**) Schematic illustration of the process for producing CNFs from brown seaweed residues. (**B**) X-ray diffraction curves of CNFs. (**C**) FTIR curves of CNFs. (**D**) Particle size of CNFs. (**E**) Zeta potential of CNFs. (**F**) Contact angles of CNFs. Different letters (a–c) indicate significant differences between groups (*p* < 0.05).

Cellulose crystallinity critically affects the suspension stability, Pickering emulsification performance, and thermal stability of emulsions. The microcrystalline cellulose, derived from natural cellulose by the hydrolysis with strong acids to remove a portion of the amorphous regions, exhibits a high degree of crystallinity, with a crystallinity index of 74.96 ± 0.74% as indicated in Table 1. As shown in Figure 1B, the presence of crystalline peaks at 22° and weaker peaks around 14° and 16° suggests that cellulose samples exhibit a typical cellulose I structure [20]. Moreover, both crystalline and amorphous domains could be observed in the X-RD profiles of present samples. The results indicated that shear and impact forces of high-pressure homogenization could not change the crystal type of cellulose [21].

In addition, the crystallinity index of the cellulose sample is shown in Table 1. The crystallinity index values of homogenized samples were lower than those of Microcrystalline Cellulose (74.96 ± 0.74) and untreated cellulose (65.23 ± 2.96). The shearing and impacting forces of high-pressure homogenization as described in Figure 1B might support such reduced crystallinity index values. With the increasing pressure of homogenization, cellulose treated at a homogenization pressure of 105 MPa exhibited a lower crystallinity index than cellulose treated at 140 MPa. The results might indicate the breaking of hydrogen bonds between cellulose molecules and be attributed to the collapse of some crystalline structure [22,23].

### 2.3. Effect of Homogenization Pressure on Chemical Construction of Brown Seaweed Cellulose

Figure 1C illustrates the FTIR spectra of CNFs. Although some shifts in peak position were shown within specific bands, the homogenized CNFs display similar FTIR spectral patterns. The peaks at 3349 cm^−1^ and 1165 cm^−1^ are the free O-H stretching vibrations of the hydroxyl group and the C-O-C vibrations of the pyranose ring. The peaks at 2896 cm^−1^ and 1426 cm^−1^ are the C-H stretching and bending bands of the -CH_2_ group. The band at 895 cm^−1^ corresponds to the vibrations of the *β*-(1,4)-glycosidic linkage. All samples retain the characteristic functional groups of cellulose, such as the above vibrations of C-O-C vibrations of the pyranose ring and the *β*-(1,4)-glycosidic, which demonstrates that high-pressure homogenization does not change the functional groups of cellulose. This is consistent with the structural characteristics reported for bacterial cellulose, further confirming the stability of the cellulose chemical structure during the treatment process [24]. The results suggested that the extracted product is cellulose, which offers dependable material for further structural analysis and applications in Pickering emulsion.

### 2.4. Effect of Homogenization Pressure on Particle Size of Cellulose Nanofibers

The hydrogen bonds in cellulose molecules might break by high-pressure homogenization, reducing the particle size and being attributed to fibrillation. As shown in Figure 1D, the particle size of untreated cellulose (H-0) was 369 nm with a non-uniform distribution, spanning between 200 and 800 nm. With the increasing pressure of homogenization, the low average particle size of the samples could be observed. The profile of the H-105 showed the two peaks at 87 nm and 295 nm, which indicated the formation of CNFs. Moreover, the smallest particle size was the H-140 CNFs with 63 nm, although the higher peak at 220 nm might suggest the bigger CNFs were still distributed in the samples. A similar result might be supported by Chen M et al., which reported wood pulp cellulose nanocrystals have mean lengths and heights of 100–300 nm and 3–5 nm, and cellulose nanocrystals tend to form lateral aggregates and multi-particle clusters because of strong interparticle hydrogen bonds [25]. The results indicated that high-pressure homogenization could obviously reduce the particle size of cellulose.

### 2.5. Effect of Homogenization Pressure on Zeta Potential of Cellulose Nanofibers

The double electric layer structure of CNFs in the aqueous phase is attributed to the negative Zeta potentials as shown in Figure 1E. The Zeta potential of untreated nanocellulose (H-0) is the lowest (−17.53 ± 0.70 mv) when compared with homogenized samples. The reason might be that the high-pressure homogenization process facilitates the stirring of the suspension, which offers a chance for nanocellulose to come into contact with oxygen, resulting in the oxidation of some particles and the production of additional negative charges [26]. Such an increase in charges on the CNFs might facilitate the electrostatic repulsion and increase the stability in the aqueous system of Pickering emulsion. With the increasing pressure from 35 to 140 MPa, the significatively increased (*p* < 0.05) negative charges could be observed in CNFs. The absolute zeta potential of nanocrystalline cellulose suspensions from palm fibers extracted using acid hydrolysis high-pressure homogenization also increased similarly, which were from −13.4 mV to −60.6 mV [27].

### 2.6. Effect of Homogenization Pressure on Contact Angle of Cellulose Nanofibers

The contact angle was measured to assess the wettability of the CNFs with different homogenized pressures. As shown in Figure 1F, all CNFs showed a hydrophilic angle that was less than 90°. The H-0 CNFs showed the biggest contact angle of 52.43 ± 2.62°. With the increasing homogenized pressure from 35 to 140 MPa, the contact angles of CNFs significatively increased (*p* < 0.05) to 51.40 ± 2.57°. Such results might be because intense homogenization pressures lead to the exposure of more amorphous and hydrophobic surfaces [28] and weaken the wettability of CNFs [29]. Enhanced hydrophobicity allows for more efficient adsorption of CNF particles at the oil/water interface, which is beneficial for the stabilization of oil-in-water Pickering emulsion [30].

### 2.7. Effect of Homogenization Pressure on Microstructure of Cellulose Nanofibers

**Figure 2:**
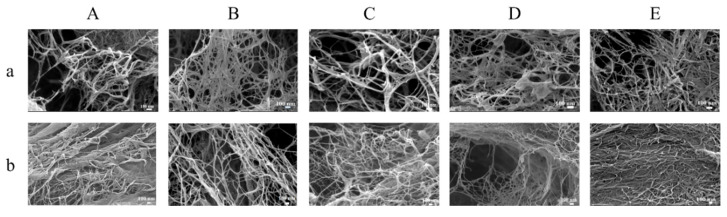
SEM image of CNFs homogenized with different pressures. (**a**) 50,000× and (**b**) 30,000×. (**A**) H-0, (**B**) H-35, (**C**) H-70, (**D**) H-105, and (**E**) H-140.

The morphology of the CNFs was observed by the SEM and shown in Figure 2. The crosslinked networks formed by cellulose were distributed in all samples. The images showed that the irregular dendritic-like structures of H-0 (Figure 2A) became short and thin fibers (Figure 2B–E) after the high-pressure homogenization. The H-105 and H-140 CNFs showed a more uniform size distribution. Such uniform crosslinked networks, which are formed by CNFs, might supply high steric hindrance in the continuous phase and enhance the stability of the Pickering emulsion.

### 2.8. Effect of Homogenization Pressure on Thermogravimetric Stability of Cellulose Nanofibers

**Figure 3:**
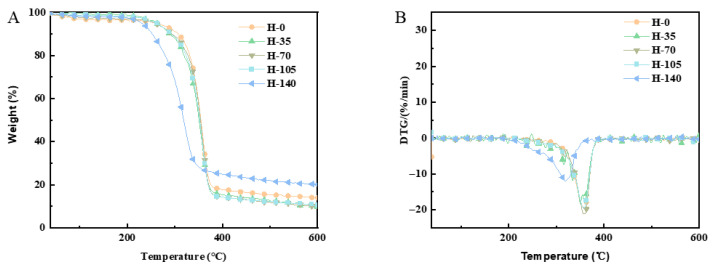
Thermal curves of CNFs homogenized with different pressures. (**A**) Thermogravimetry (TG) curves. (**B**) Derivative thermogravimetry (DTG) curves.

As shown in Figure 3A, thermogravimetric analysis was used to evaluate the effect of homogenization on the thermal stability of cellulose. Cellulose with smaller particle sizes not only lowers the thermal decomposition temperature but also promotes more complete degradation. Sample thermal stability was characterized by the degradation temperature corresponding to maximum degradation rate. H-140 had a lower thermal decomposition temperature than H-105, indicating that H-105 showed better thermal stability.

In the Figure 3B, three mass loss stages occurred during heating. The first stage (around 37–298 °C) was attributed to free water evaporation and low-molecular-weight compound degradation, as evidenced by a small mass loss peak in all samples. The second stage (around 298–400 °C) corresponded to cellulose pyrolysis, and the third stage (around 400–600 °C) was due to residual volatile release. High-pressure homogenization reduced the pyrolysis range compared with untreated cellulose. After the entire thermal decomposition process, the mass loss of cellulose subjected to 0 MPa homogenization pressure was 85.25%, while the mass loss of cellulose subjected to 140 MPa pressure was 79.63%, a reduction of 5.62% compared with 0 MPa. High-pressure homogenization reduces cellulose particle size and increases specific surface area. It also introduces structural defects and amorphous regions, lowering cellulose thermal stability [31]. Consequently, during thermal decomposition, there was greater mass loss and less residual content.

### 2.9. Encapsulation Efficiency of Fucoxanthin in Pickering Emulsion

**Figure 4:**
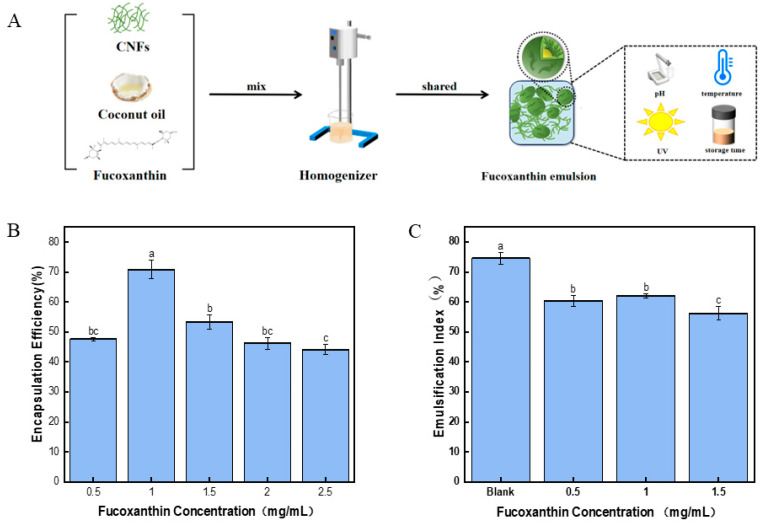
Preparation and evaluation of fucoxanthin emulsions stabilized by CNFs. (**A**) Schematic representation of the process for preparing fucoxanthin emulsion stabilized by CNFs. (**B**) Encapsulation efficiency. (**C**) Centrifugal stability. Different letters (a–c) indicate significant differences between groups (*p* < 0.05).

The encapsulation efficiency was a critical parameter for evaluating the performance of active compounds in Pickering emulsion delivery systems. In addition, the encapsulation efficiency could significantly impact the stability of Pickering emulsion. As shown in Figure 4B, with the increasing fucoxanthin concentration, the encapsulation efficiency of fucoxanthin in the Pickering emulsion initially increases and then decreases. Such results were supported by the similar encapsulation efficiency of curcumin [32], and the concentration of curcumin ranged from 300–800 mg/mL. In the Pickering emulsion with 1.0 mg/mL fucoxanthin, the encapsulation efficiency of the Pickering emulsion was the highest (70.84 ± 3.91%). However, the results indicated that Pickering emulsion with the highest concentration of fucoxanthin (2.5 mg/mL) provided the lowest encapsulation efficiency, which was 44.18 ± 1.64%. A recent study showed that the ultrasound encapsulation of fucoxanthin into royal-jelly exosomes peaked at a 1:2 mass ratio with 65% loading efficiency [33]. The result was significantly lower than the present 1.0 mg/mL fucoxanthin emulsion. Increasing the concentration of fucoxanthin might decrease the interfacial tension of the emulsion and decrease the stability of the emulsion [12]. The encapsulation efficiency of the fucoxanthin might affect the antioxidant activity of the Pickering emulsion and provide a benefit function in the delivery system in vivo. With regard to the above results, the Pickering emulsion with 0.5 mg/mL, 1 mg/mL, and 1.5 mg/mL fucoxanthin, which provided high encapsulation rates, was used for further study.

### 2.10. The Stability of the Fucoxanthin Emulsion

Centrifugation stability can evaluate the stability of the Pickering emulsions during long-term storage [34]. Figure 4C illustrates the emulsification index of centrifugated Pickering emulsions at different concentrations of fucoxanthin. The results indicated that the Pickering emulsion with fucoxanthin showed a lower emulsification index than those without fucoxanthin. Such results might suggest that fucoxanthin could weaken the centrifugation stability of the Pickering emulsion. A high concentration of fucoxanthin might disrupt the stability of the gel-like networks in emulsion and contribute to reducing emulsion stability [35]. The Pickering emulsion with 1.5 mg/mL of fucoxanthin had the lowest emulsification index of 56.25 ± 2.31%, which was significantly lower than those of the control (74.58 ± 1.91%). The 1.0 mg/mL fucoxanthin emulsion showed a high emulsification index of 62.10 ± 0.72% after centrifugation, which was supported by the high encapsulation efficiency as described in Section 2.9.

**Figure 5:**
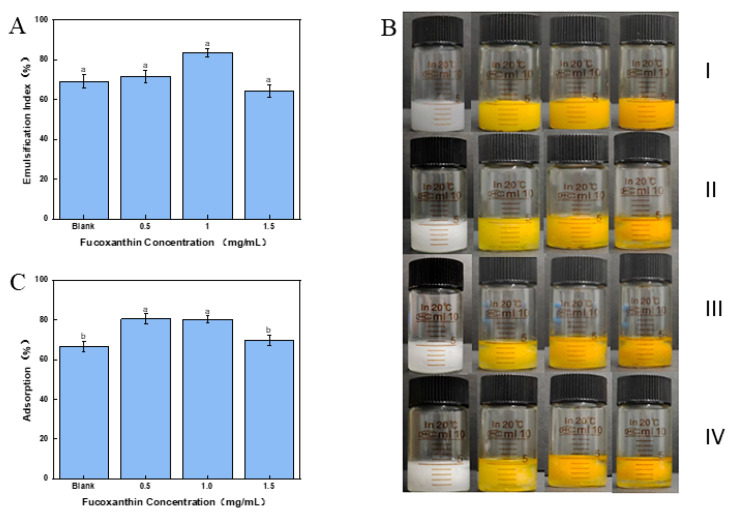
Freeze–thaw stability and interfacial adsorption of fucoxanthin emulsions. (**A**) Emulsification index after freeze–thaw cycles. (**B**) Appearance morphology of fucoxanthin emulsion during three freeze–thaw cycles. (**C**) The relative interfacial adsorption. Different letters (a,b) indicate significant differences between groups (*p* < 0.05).

Freeze–thaw stability could evaluate the stability of the Pickering emulsion in marine foods and drugs with freezing processing. Figure 5A,B show the appearance of an emulsification index of the Pickering emulsion with three freeze–thaw cycles. Although oil separation at the bottom was observed in the Pickering emulsion with 1.5 mg/mL fucoxanthin, other present Pickering emulsions all maintained uniform emulsification after one freeze–thaw cycle. The ice crystals formed during the freezing process were possibly consequent upon such results, leading to the aggregation between Pickering emulsion droplets and resulting in phase separation [36]. Furthermore, no significant difference was there in the present Pickering emulsions after three freeze–thaw cycles (Figure 5A). Such results might suggest that fucoxanthin could not weaken the stability of the emulsion, which might benefit the efficiency of the drug delivery system [37]. The 1.0 mg/mL fucoxanthin emulsion remained at 83.33 ± 2.12% emulsification index after three freeze–thaw cycles, showing potential application in freeze-processing foods, such as ice creams.

### 2.11. Interfacial Adsorption Amount of the Cellulose Nanofibers in Fucoxanthin Emulsion

The thick interfacial layer formed by emulsifiers provides effective steric hindrance and confers long-term stability in emulsions [38]. As shown in Figure 5C, the effect of fucoxanthin on the relative interfacial adsorption of nanocellulose within the Pickering emulsion was evaluated. The interfacial adsorption of nanocellulose in the Pickering emulsion with 0.5 and 1.0 mg/mL fucoxanthin was significantly increased relative to the blank control (*p* < 0.05). With the increasing fucoxanthin concentration from 0.5 to 1.5 mg/mL, the interfacial adsorption of nanocellulose was obviously reduced. The result was supported by the encapsulation efficiency of fucoxanthin within the Pickering emulsion. A comparable conclusion was confirmed with Pickering emulsions encapsulated with curcumin [39], which provided enhanced gel strength and superior stability compared with those without curcumin. Such results might confirm that 0.5 to 1.5 mg/mL fucoxanthin promoted developed CNF interfacial films upon the Pickering emulsion droplets. In view of the characteristics of CNFs with high-pressure homogenization, the high interfacial adsorption of CNFs might provide enhanced thermogravimetric stability and gel strength of the Pickering emulsion. In conclusion, the Pickering emulsion with a fucoxanthin concentration of 1.0 mg/mL was selected for subsequent measurements.

### 2.12. Effect of pH Value on the Stability of Fucoxanthin Emulsion

**Figure 6:**
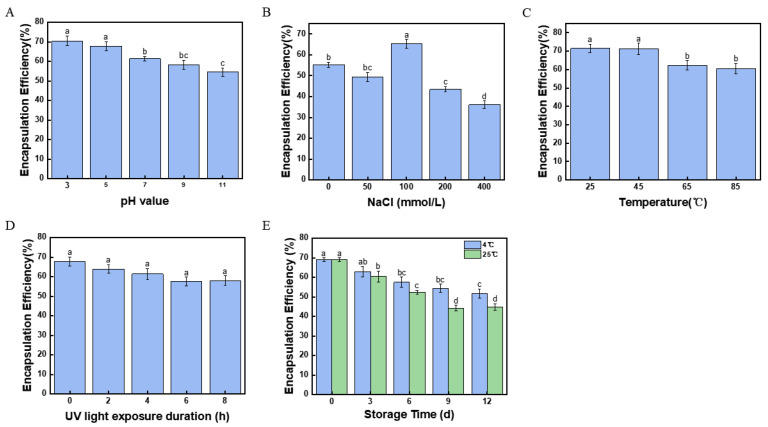
The effect of different conditions on the encapsulation efficiency of fucoxanthin in Pickering emulsion. (**A**) The pH value. (**B**) The salinity. (**C**) The temperature. (**D**) The UV light exposure duration. (**E**) The storage time. Different letters (a–d,ab,bc) indicate significant differences between groups (*p* < 0.05).

Processing and storage factors, such as pH value, salinity, temperature, UV light exposure duration, and storage time, markedly impact the stability and biological activity of emulsion [40,41]. The surface charge of solid particles and the degree of ionization of functional groups could be controlled by the pH value and impact the stability of the emulsion [42]. As shown in Figure 6A, with the increasing pH value, the encapsulation efficiency of fucoxanthin significantly decreased (*p* < 0.05). At pH 3.0 and 5.0, the encapsulation efficiencies of fucoxanthin were 70.48 ± 2.62% and 67.70 ± 3.44%, respectively. In the study of the pH stability of astaxanthin emulsions, the encapsulation efficiency of the astaxanthin emulsion was only 55.36% at pH 5.0 [43], which was significantly lower than that of the fucoxanthin emulsion. The results confirmed that acidic conditions offered high stability of the fucoxanthin emulsion. Furthermore, the CNFs with high-pressure homogenization showed the potential capability to preserve fucoxanthin upon the water–oil interface in an acidic Pickering emulsion.

### 2.13. Effect of Salinity on the Stability of Fucoxanthin Emulsion

The addition of salinity in Pickering emulsion provides electrostatic shielding for the negative charge of nanocellulose. By reducing particle repulsion, the salinity improves the particle distribution at the oil–water interface, thereby enhancing emulsion stability [44]. As shown in Figure 6B, the encapsulation efficiency of fucoxanthin in the Pickering emulsion with 100 mmol/L NaCl reached 65.20 ± 2.18%, which was significantly higher than other Pickering emulsions. However, a negative effect could be observed on the encapsulation efficiency of fucoxanthin with the addition of NaCl in Pickering emulsion. Although the addition of NaCl could strengthen emulsion stability to a certain degree, high salinity concentrations may damage the interfacial structure of droplets and lead to the degradation of fucoxanthin [45].

### 2.14. Effect of Temperature on the Stability of Fucoxanthin Emulsion

Thermal processing is widely used in food production and applications. However, high temperature weakens the stability of the Pickering emulsion. For the Pickering emulsions’ encapsulated thermolabile active compounds, strict temperature is essential for thermal processing. The effects of temperature on the encapsulation efficiency of fucoxanthin emulsion were measured. As shown in Figure 6C, with the increasing temperature from 25 °C to 85 °C, the encapsulation efficiency of fucoxanthin in Pickering emulsion was obviously decreased. Although the encapsulation efficiency of fucoxanthin was 60.49 ± 2.80% at 85 °C, which was significantly lower than that at 25 °C (71.51 ± 2.32%), the 85 °C value still maintained a high encapsulation efficiency. H. Park [46] reported similar emulsion stabilized by protein-loaded poly (lactic-co-glycolic acid) microspheres with denoted 60.66% encapsulation efficiency at 45 °C. The thermal insulation provided by the CNF barrier at the oil–water interface potentially stems from such high encapsulation efficiency and protecting the bioactivity of the fucoxanthin [47].

### 2.15. Effect of UV Exposure on the Stability of Fucoxanthin Emulsion

The functional groups in fucoxanthin molecular, such as hydroxyl, 5,6-epoxy, and carbonyl groups, supply a highly susceptible structure for oxidation and degradation. As shown in Figure 6D, with the UV irradiation time extending to 8 h, no significant difference (*p* < 0.05) could be observed in the encapsulation efficiency of fucoxanthin. Although there was a slight decrease in numerical value, the high encapsulation efficiency remained in fucoxanthin emulsion. The well-formed nanocellulose network in the Pickering emulsion might contribute to such results, and protect fucoxanthin from UV exposure. Similarly, the Pickering emulsions stabilized by *kefiran* nanoparticles and Tween-80 retained about 60% and 50% of curcumin after 270 min of UV exposure [48],which was lower than the present fucoxanthin emulsion at 4 h of UV exposure. The results might support the conclusion reported by Yeongsheng L et al., who affirmed that Pickering emulsions appear to be a more effective system for protecting curcumin from UV degradation [49].

### 2.16. Effect of Storage Time on the Stability of Fucoxanthin Emulsion

Fucoxanthin is susceptible to temperature and storage time, limiting the application of Pickering emulsion in food. For evaluating the stability of fucoxanthin emulsion, the present Pickering emulsions were stored at 4 °C and 25 °C for 12 days. The encapsulation efficiency of fucoxanthin was assessed at regular intervals (Figure 6E). The results indicated that the encapsulation efficiency of fucoxanthin declined in both 4 °C and 25 °C Pickering emulsion during the 12 days of storage. The encapsulation efficiencies of fucoxanthin in Pickering emulsion kept at 4 °C and 25 °C were 51.63 ± 2.43% and 44.79 ± 1.58%, respectively. The Brownian motion among droplets during storage was possibly consequent upon such results, enlarging droplet size and diminishing with aggregation post-collision [50]. In addition, the coconut oil in the Pickering emulsion may solidify between 14 °C and 25 °C [51], thereby strengthening the stability of the Pickering emulsion. Moreover, the solidified coconut oil in the Pickering emulsion might supply a higher retention rate of fucoxanthin when stored at 4 °C compared with 25 °C. Furthermore, such results might suggest that low-temperature storage of the Pickering emulsion can retard the degradation of fucoxanthin, benefitting the bioactivity of fucoxanthin in the delivery system of marine drugs.

### 2.17. Effect of pH Value on the Antioxidant Activity of Fucoxanthin in Pickering Emulsions

Food thermal processing-related unstable factors such as temperature, pH, light, and salinity significantly affect the bioactivity of the fucoxanthin in Pickering emulsion. Supported by the enhanced stability described in Section 2.12, Section 2.13, Section 2.14, Section 2.15 and Section 2.16, the bioactivity of fucoxanthin is crucial for the delivery system of marine drugs. For the evaluation of the antioxidant activity, the ABTS cationic and DPPH free radical scavenging capacity of the fucoxanthin in Pickering emulsion with different processing and storage factors were measured, respectively.

**Figure 7:**
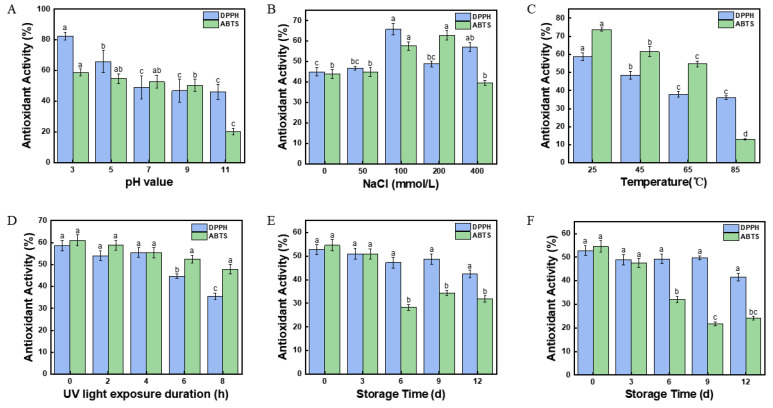
The effect of different conditions on antioxidant activity of fucoxanthin emulsion. (**A**) The pH value. (**B**) The salinity. (**C**) The temperature. (**D**) The UV light exposure duration. (**E**) The storage time at 4 °C. (**F**) The storage time at 25 °C. Different letters (a–d,ab,bc) indicate significant differences between groups (*p* < 0.05).

As shown in Figure 7A, with the increasing pH value from 3.0 to 11.0, the DPPH free radical scavenging rate in Pickering emulsion was obviously decreased. At pH 3.0, the DPPH free radical scavenging rate of the fucoxanthin was 82.45 ± 2.46%, which was significantly higher (*p* < 0.05) than other Pickering emulsions. Such results might be related to the encapsulation efficiency of fucoxanthin described in Section 2.12. The DPPH free radical scavenging rate of fucoxanthin in Pickering emulsion increased with the increasing encapsulation efficiency. The results might suggest that an increased concentration of hydrogen ions could disrupt the antioxidant activity of fucoxanthin in emulsion [52]. A similar decrease could be observed in the ABTS cationic free radical scavenging rate of the fucoxanthin emulsion. At pH 3.0, the ABTS cationic free radical scavenging rate was highest (58.71 ± 2.27%). This value was also higher than the 41.17% reported by Saravana, P. S et al. [53] in fucoxanthin-rich oil nano emulsions, where thick interfacial layers limited radical accessibility. With the consideration of the encapsulation efficiency of fucoxanthin described in Section 2.12, the CNFs interface upon the emulsion droplets effectively weakened the destruction of pH on fucoxanthin. In addition, the high interfacial adsorption of 1.0 mg/mL CNFs (Figure 5C) might prevent the unstable effects of fucoxanthin emulsion.

### 2.18. Effect of Salinity on the Antioxidant Activity of Fucoxanthin in Pickering Emulsions

As shown in Figure 7B, the salinity in Pickering emulsion showed an obvious effect on the antioxidant activity of fucoxanthin in Pickering emulsion. With the increasing salinity from 50 to 400 mmol/L, the ABTS cationic and DPPH free radical scavenging rate was increased at first and then decreased. In the fucoxanthin emulsion with 100 mmol/L NaCl, the DPPH free radical scavenging rate of the fucoxanthin was 82.45 ± 2.46%, which was the highest in the present Pickering emulsions. The results might be supported by the high encapsulation efficiency of fucoxanthin in Pickering emulsion at 100 mmol/L (Figure 6B). The high encapsulation efficiency might be beneficial for the antioxidant activity of the fucoxanthin in Pickering emulsion. Moreover, in the fucoxanthin emulsion with 200 mmol/L NaCl, the highest ABTS cationic radical scavenging rate of the fucoxanthin in the present Pickering emulsions could be observed (62.83 ± 2.35%). The result might indicate that the high concentrations of NaCl might disrupt the stability of the fucoxanthin emulsion, leading to an irregular antioxidant activity of fucoxanthin [54].

### 2.19. Effect of Temperature on the Antioxidant Activity of Fucoxanthin in Pickering Emulsions

As shown in Figure 7C, the results indicated that temperature significantly affected (*p* < 0.05) the antioxidant activity of fucoxanthin in Pickering emulsion. With the increasing temperature from 25 °C to 85 °C, the DPPH free radical scavenging rate of fucoxanthin in Pickering emulsion was obviously decreased. In the Pickering emulsion at 25 °C, the DPPH free radical scavenging rate of the fucoxanthin was 58.69 ± 2.12%, significantly higher (*p* < 0.05) than other Pickering emulsions. The DPPH free radical scavenging rate was higher than the rate of lutein after 1 h of treatment at 40 °C (53.53%) reported by Ishani Bhat et al. [55]. However, when the temperature was increased to 85 °C, the DPPH free radical scavenging rate of the fucoxanthin emulsion was 35.91 ± 1.11%. The high temperature might degrade fucoxanthin in the Pickering emulsion, thereby reducing the antioxidant activity of fucoxanthin. The ABTS cationic radical scavenging rate of fucoxanthin showed a similar result to the DPPH free radical scavenging rate. The Pickering emulsion at 25 °C showed the highest ABTS cationic radical scavenging rate with 73.70 ± 0.86%. Such results might be supported by the encapsulation efficiency of fucoxanthin described in Section 2.14. Furthermore, the CNF interface upon the emulsion droplets might supply a high encapsulation efficiency of fucoxanthin [56], thereby preventing the fucoxanthin from degradation with thermal processing.

### 2.20. Effect of UV Irradiation on the Antioxidant Activity of Fucoxanthin in Pickering Emulsions

Figure 7D shows the effect of the UV irradiation on the antioxidant activity of fucoxanthin in Pickering emulsion. With the increasing time of the UV irradiation from 2 to 8 h, the antioxidant activity of fucoxanthin in Pickering emulsion was obviously decreased. Compared with the Pickering emulsion without UV irradiation (58.68 ± 2.32%), the DPPH free radical scavenging ability was decreased by 23.23% after 8 h (35.46 ± 1.47%). The ABTS cationic radical scavenging rate showed a similar decrease to the above results. With the 8 h UV irradiation on Pickering emulsions, the ABTS cationic radical scavenging rate had been as low as 47.85 ± 2.18%. Such a result might prove that prolonged UV irradiation on the Pickering emulsion leads to the degradation of fucoxanthin [57], which provides a potential strategy for the storage of fucoxanthin emulsion stabilized by CNFs.

### 2.21. Effect of Storage Time on the Antioxidant Activity of Fucoxanthin in Pickering Emulsions

The effect of storage time on the antioxidant activity of fucoxanthin in Pickering emulsion is shown in Figure 7E,F. When stored at 4 °C, the ABTS cationic and DPPH free radical scavenging obviously decreased with increasing time. Compared with the fresh Pickering emulsion (52.73 ± 2.08%), the DPPH free radical scavenging rate of fucoxanthin was decreased by 10.36% after 12 day storage (42.37 ± 1.59%). The study reported by Robles-García, M. Á et al. [58] found that yogurt enriched with 10% nano capsules stored at 4 °C for 14 days had a DPPH free radical scavenging rate of 51.3%. This demonstrates good antioxidant stability. The ABTS cationic radical scavenging rate showed a similar reduction by 22.64% after 12 day compared with the fresh Pickering emulsion (54.52 ± 2.45%). In addition, the antioxidant activity of fucoxanthin in the Pickering emulsion stored at 25 °C was similar to that at 4 °C. When stored at 25 °C, the ABTS cationic and DPPH free radical scavenging rates of the fucoxanthin were both obviously decreased (Figure 7F). The double bonds and oxygen groups of the fucoxanthin might contribute to such degradation [59]. Moreover, after 12 days of storage at 4 °C, the fucoxanthin emulsion had a higher antioxidant activity than that stored at 25 °C. Therefore, the antioxidant activity of the Pickering emulsion was better maintained when stored at low temperatures. Furthermore, as a protective barrier in Pickering emulsions, the CNFs might prevent the fucoxanthin from oxidation.

This study has certain limitations. The correlation between the structure and interface properties of the CNFs in Pickering emulsions was not extensively explored. These factors are crucial for industrial applications. In addition, further discussion on industrial implications is needed, especially regarding compatibility with processing equipment and scalability of the emulsion preparation process. The future study will involve collaboration with seaweed processing industries to conduct a comprehensive industrial analysis, ensuring the developed emulsions can be effectively translated into large-scale production. Moreover, cytotoxicity and biocompatibility assessments will be performed or planned to support pharmaceutical applications in the next study.

## 3. Materials and Methods

### 3.1. Materials

The raw material used in this study was the discarded residue from the extraction of alginate, provided by Qingdao Mingyue Group (Qingdao, China). The glucan content in the industrial waste residue of brown seaweed was 94.52 ± 7.90%, the ash content was 2.63 ± 0.53%, and the moisture content was 90.06 ± 0.07%. The fucoxanthin (analytical grade) was purchased from Qingdao Mingyue Group (Qingdao, China). The coconut oil (food grade) was purchased from Wenchang Dongjiao Coconut Processing Professional Cooperative (Hainan, China).

The anhydrous ethanol, glacial acetic acid, tertiary butanol, potassium persulfate, copper ethylenediamine solution, calcium carbonate potassium bromide sodium chlorite, sodium chloride, glucose, and sodium hydroxide were all analytical grade and were purchased from Macklin Co., Ltd. (Shanghai, China). The 2,2-diphenyl-1-picrylhydrazyl (DPPH) and 2,2′-azino-bis (3-ethylbenzothiazoline-6-sulfonic acid) diammonium salt (ABTS) used in the present study were all analytical grade and purchased from Sigma-Aldrich Co., Ltd. (Alexandria, VA, USA). The glucose determination kit was purchased from Abcam Co., Ltd. (ab65333, Shanghai, China). The concentrated sulfuric acid was analytical grade and was also purchased from Fuyu Fine Chemical Co., Ltd. (Beijing, China).

### 3.2. Isolation of Brown Seaweed Cellulose

First, brown seaweed was isolated from brown seaweed industrial waste residue using a previously reported procedure [18]. Briefly, the industrial waste residue of brown seaweed powder was treated with methanol, sodium chloride, sodium hydroxide, and hydro-chloric acid to remove the nontarget components. Then the mixture was washed in deionized water until pH 7.0 to obtain cellulose. The wet sample was then rinsed with t-butanol, freeze-dried, and stored in a desiccator for structural analysis and enzymatic hydrolysis experiments.

### 3.3. Preparation of the Cellulose Nanofibrils (CNFs)

The CNFs were prepared by high-pressure homogenization. Briefly, the cellulose isolated from brown seaweed was diluted into 50 mg/mL with deionized water (pH 7.0). Then the cellulose was 35, 70, 105, and 140 MPa homogenized by a high-pressure homogenizer (AH-BASIC, GEA Niro Soavi, Parma, Italy) 10 times. The final CNFs were stored at 4 °C for study.

### 3.4. Determination on the Polymerization Degree of the CNFs

The determination of the polymerization degree of the CNFs was carried out according to a previously reported method [18]. Briefly, the cellulose sample (40 mg) was mixed with 20 mL of 0.5 mol/L copper ethylenediamine solution with magnetic stirring (SH23-2, Meiyingpu, Shanghai, China) at 25 °C. A 15 mL portion of the solution was pipetted into an Ubbelohde viscometer. After being kept in a 25 °C water bath for 30 min, the efflux time of the solution was measured. The measurements were performed in triplicate, and the average value was reported. The polymerization degree of the sample was calculated using Equation (1).

(1) $$Polymerization\;degree=\left[\eta \right]\times 190$$

where $\left[\eta \right]$ represents the cellulose viscosity.

### 3.5. X-Ray Diffraction Analysis of the CNFs

Cellulose samples were analyzed using an X-ray diffraction (XRD) instrument (XPert PRO, PANalytical B.V., Almelo, Netherlands) equipped with radiation (λ = 0.1542 nm). The analysis was generated at 40 kV and 40 mA. Diffraction intensity was measured at diffraction angles (2θ) varying from 5° to 60° at a scanning rate of 2°/min. The crystallinity index (CrI) of the samples was calculated according to the diffraction patterns using Equation (2):

(2) $$CrI=\frac{\left(I_{c}-I_{a}\right)}{I_{c}}\times 100\%$$

where I_c_ represents the diffraction intensity of the crystalline region, and I_a_ represents the diffraction intensity of the amorphous region.

### 3.6. Fourier-Transform Infrared Spectroscopy (FTIR) Analysis of the CNFs

The CNFs samples were ground with KBr at a mixing ratio of 1:100 and pressed into discs with a hydraulic press (FW-SA, Botianshengda, Tianjin, China). Then, the analysis was performed with a FTIR Spectrometer (NICOLET iS50, Thermon Scientific, MA, USA). The FTIR spectrum was acquired in transmittance mode with a wave number range of 4000 to 400 cm^−1^, accompanied by a resolution of 4 cm^−1^ with 32 scans.

### 3.7. Determination on Particle Size and Zeta Potential of the CNFs

The particle size of CNFs was measured using a nano laser particle size analyzer (Nano-ZS90, Malvern, Jessup, UK). The CNF samples were analyzed with a 90° incident angle and 1.330 refractive index at 25 °C. The results were analyzed using Zeta sizer Software (Malvern Panalytical 3.30, Malvern, Jessup, UK).

In addition, the Zeta potential of the CNFs samples was determined using the same Zeta potential analyzer (Nano-ZS90, Malvern, Jessup, UK). The dispersions of samples were diluted into 1.38 μg/mL. Then, 1.0 mL of the sample (pH 7.0) was measured at 25 °C. The results were analyzed using Zeta sizer Software (Malvern Panalytical 3.30, Malvern, Jessup, UK).

### 3.8. Contact Angle Analysis of the CNFs

The oil/water interface characteristics of the CNFs were evaluated by the contact angle using a drop shape analyzer (DSA 100, KRÜSS, Hamburg, Germany). First, the freeze-dried samples were coated onto microscope slides and dried in a constant temperature and humidity chamber (HWS-80B, Hongnuo, Tianjin, China) to form a thin film. After equilibrium, a drop of Milli-Q water (5 μL) was slowly excluded from the syringe and quickly attached to the surface of the samples. The shapes of the water drops were recorded by a camera. The contact angles were numerically solved by the Advance software 1.7.2.2 (ADVANCE, KRÜSS, Hamburg, Germany).

### 3.9. Morphology Observation of the CNFs

The morphology of the CNFs was observed by scanning electron microscope (SEM) (JSM-7800F, HITACHI, Tokyo, Japan) as described by Li et al. [60] with minor modifications. Briefly, the freeze-dried CNFs samples were ground, and the powder was put on a specimen stage equipped with conductive adhesive. After coating with a gold-palladium alloy for 60 s at 10 mA, the CNFs samples were observed by an SEM at 30 K and 50 K magnification times. The images were processed using an SEM series software V5.07 (Zeiss Smart SEM, HITACHI, Tokyo, Japan).

### 3.10. Thermogravimetric Analysis of the CNFs

Thermogravimetric analysis (TG) and differential thermal analysis (DTG) were performed using a simultaneous thermal analyzer (STA449F3, NETZSCH, Selb, Germany). Twenty milligrams of cellulose samples under different homogenization pressures were analyzed from 37 °C to 600 °C. The test was conducted at a heating rate of 50 mL/min and 10 °C/min.

### 3.11. Preparation of the Fucoxanthin Emulsion Stabilized by the CNFs

The fucoxanthin emulsion was prepared based on the optimal results of the orthogonal experiment (Table S2). The CNFs suspensions of 11.0 mg/mL were dispersed in deionized water and adjusted to pH 7.0. Then, different concentrations (0.5, 1.0, 1.5, 2.0, and 2.5 mg/mL) of fucoxanthin were dissolved with coconut oil and added into the CNF suspensions at 10% (*v*/*v*) of final Pickering emulsions. Next, the mixture was homogenized by a homogenizer (THF500-G, Tuohe, Shanghai, China) at 10,000 rpm for 120 s. Finally, the Pickering emulsion samples were stored at 4 °C for 30 min to obtain the fucoxanthin emulsion and were recorded by a digital camera (COOLPIX P900s, Nikon, Tokyo, Japan).

### 3.12. Determination on Encapsulation Efficiency of Fucoxanthin in Pickering Emulsion

The encapsulation efficiency of fucoxanthin in Pickering emulsion was determined according to the previous study reported by Zhe et al. [61], with minor modification. Briefly, one gram of fucoxanthin emulsion with varying fucoxanthin concentrations (0.5, 1.0, 1.5, 2.0, and 2.5 mg/mL) was added into 5 mL anhydrous ethanol for the extraction of fucoxanthin. After thoroughly mixing, the mixture was centrifuged at 10,000 rpm for 10 min. Then, the supernatant was diluted into 10 mL, and the absorbance of the samples was measured at 450 nm. A standard curve of fucoxanthin (y = 0.045x + 0.0623, 0 < x < 1.0 μg/mL, R^2^ = 0.997) was used for the analysis of the encapsulation rate in the fucoxanthin emulsion, which was calculated with the following Formula (3):

(3) $$Encapsulation\;Efficiency\left(\%\right)=\frac{M_{i}}{M_{e}}\times 100\%$$

where M_i_ represents the total amount of fucoxanthin in the initial Pickering emulsion, and M_e_ represents the total amount of fucoxanthin encapsulated in the Pickering emulsion.

### 3.13. Analysis on Centrifugal Stability of the Fucoxanthin Emulsion

The emulsification index was investigated to evaluate the centrifugation stability of the Pickering emulsions. According to the centrifugal method reported by Bai et al. [62], 5 g of the fucoxanthin emulsion was centrifuged with the centrifugal machine (HH500-18G, Huiyi, Shanghai, China) at 5000 r/min for 2 min at 25 °C. The total height of the Pickering emulsion ($H_{w}$) and the height of the gel-like Pickering emulsion ($H_{e}$) after centrifugation were measured by a vernier caliper (DL91150, Deli, Ningbo, China). The results were reported as the emulsification index, which was calculated based on Equation (4):

(4) $$Emulsifician\;Index=\frac{H_{e}}{H_{w}}\times 100\%$$

### 3.14. Analysis on Freeze–Thaw Stability of the Fucoxanthin Emulsion

For the evaluation on the stability of the fucoxanthin emulsion in low-temperature storage, the freeze–thaw stability of the fucoxanthin emulsion was evaluated. First, five grams of the fucoxanthin emulsion was sealed in a glass bottle. After 12 h storage at −20 °C, the fucoxanthin emulsion was stored in a constant temperature and humidity chamber (HWS-80B, Hongnuo, Tianjin, China) at 25 °C. Such a freeze–thaw cycle was performed in triplicate, and the sample was centrifuged at 5000 r/min for 2 min at 25 °C. Finally, the freeze–thaw emulsification index was calculated based on Equation (4). The final samples after freeze–thaw were recorded by a digital camera (COOLPIX P900s, Nikon, Tokyo, Japan).

### 3.15. Determination on Interfacial Adsorption Amount of the CNFs

The interfacial adsorption of the CNFs was determined by the method reported by Li et al. [60], but there were a few modifications. Briefly, the present fucoxanthin emulsion (5 g) was centrifuged at 5000 r/min for 6 min. After the centrifugation, the CNFs were isolated from the supernatant and dried to constant weight (C_S_). Then the interfacial adsorption was calculated as Equation (5):

(5) $$Adsorption=100-\frac{C_{s}}{C_{0}}\times 100\%$$

where C_S_ represents the concentration of the CNFs in supernatant following Pickering emulsion centrifugation, and C_0_ is the initial concentration of CNFs in the aqueous phase.

### 3.16. Preparation of the Fucoxanthin Emulsion with Different pH Values

For the evaluation of the stability of the fucoxanthin emulsion in a food processing environment, the fucoxanthin emulsion was prepared in different pH values, salt ions, temperatures, and UV exposure conditions. To prepare the fucoxanthin emulsion of different pH values, the CNF suspensions were adjusted to pH 3.0, 5.0, 7.0, 9.0, and 11.0, using 0.5 mol/L HCl or NaOH. Then, the 1.0 mg/mL of fucoxanthin was dissolved with coconut oil and added into the CNF suspensions at 10% (*v*/*v*) of final Pickering emulsions. The fucoxanthin emulsion with different pH values was prepared with the method described in Section 3.11.

### 3.17. Preparation of the Fucoxanthin Emulsion with Different Salinities

To prepare the fucoxanthin emulsion with different concentrations of salinity, the CNF suspensions were added to 0, 50, 100, 200, and 400 mmol/L of NaCl and adjusted to pH 7.0. Then, the fucoxanthin emulsion with different concentrations of NaCl was prepared with the method described in Section 3.11.

### 3.18. Preparation of the Fucoxanthin Emulsion with Different Temperatures

To prepare the fucoxanthin emulsion with different temperatures, the fucoxanthin emulsion was stabilized with the method described in Section 3.11. Then, the fucoxanthin emulsion sealed in glass vials was stored at 25 °C, 45 °C, 65 °C, and 85 °C for 2 h with a digital water bath with constant temperature and humidity control (HH-8J, Langyue, Changzhou, China). The determination was immediately performed while the fucoxanthin emulsion was cooled to room temperature.

### 3.19. Preparation of the Fucoxanthin Emulsion with Different UV Light Exposure Durations

The fucoxanthin emulsion with different UV light exposure durations was stabilized with the method described in Section 3.11. The fucoxanthin emulsion was sealed in plastic centrifuge tubes and irradiated with UV light (220 V, 18 W) for durations of 0, 2, 4, 6, and 8 h. The fucoxanthin emulsion was stored at 4 °C for the next study.

### 3.20. Encapsulation Efficiency of Fucoxanthin in the Pickering Emulsion with Different Processing and Storage Factors

The fucoxanthin emulsion with different pH values, salt ions, temperature, and UV exposure were collected. According to the procedure described in Section 3.12, the encapsulation efficiency of fucoxanthin in the present Pickering emulsions was determined. Moreover, the fucoxanthin in Pickering emulsion was extracted at regular intervals for further study.

### 3.21. DPPH Free Radical Scavenging Rate of the Fucoxanthin in Pickering Emulsion

The DPPH free radical scavenging rate of the collected fucoxanthin as described in Section 3.16, Section 3.17, Section 3.18, Section 3.19 and Section 3.20 was determined according to a previously reported method [63]. Briefly, 0.3 g of the fucoxanthin emulsion was added into 1.2 mL of DPPH ethanol solution. After 30 min in the dark at 25 °C, the fucoxanthin emulsion was centrifuged at 10,000 r/min for 10 min. The supernatants were collected, and the absorbance (A_i_) of the supernatant was measured at 517 nm with a microplate reader (EPOCH2, BioTek, Winuski, VT, USA). Meanwhile, the absorbance of 0.3 g fucoxanthin emulsion with 1.2 mL of 95% ethanol solution (A_j_) and 0.3 g of 95% ethanol solution with 1.2 mL of DPPH ethanol solution was (A_0_) recorded as control. The DPPH free radical scavenging rate of the fucoxanthin emulsion was calculated using the following Formula (6):

(6) $$DPPH\;free\;radical\;scavenging\;rate\;(\%)=\left(1-\frac{A_{i}-A_{j}}{A_{0}}\right)\times 100\%$$

### 3.22. ABTS Cation Radical Scavenging Rate of the Fucoxanthin in Pickering Emulsion

The ABTS cation radical scavenging rate of the collected fucoxanthin as described in Section 3.16, Section 3.17, Section 3.18, Section 3.19 and Section 3.20 was determined according to a previously reported method [63]. After 15 min in the dark at 25 °C, the fucoxanthin emulsion was centrifuged at 10,000 r/min for 10 min. The supernatant was collected, and the absorbance was measured at 734 nm with a microplate reader (A_t_) (EPOCH2, BioTek, Winuski, VT, USA). The deionized water was used as a blank control (A_0_). The ABTS cation radical scavenging rate of fucoxanthin in the Pickering emulsion was calculated using the following Formula (7):

(7) $$ABTS\;cation\;radical\;scavenging\;rate\;(\%)=\frac{A_{0}-A_{t}}{A_{0}}\times 100\%$$

### 3.23. Statistical Analysis

Each experiment was conducted in triplicate. The mean and standard deviation of the data were calculated for each treatment. Analysis of variance (ANOVA) was carried out to determine any significant differences (*p* < 0.05) among the applied treatments with the SPSS software package (SPSS 16.0 for Windows, IBM, New York, NY, USA). The Origin 2018 software was utilized for data plotting.

## 4. Conclusions

In conclusion, the study indicated that high-pressure homogenization significantly affects the contact angle and Zeta potential of the CNFs. The CNFs with 105 MPa showed high Zeta potential and improved thermal stability. In addition, the Pickering emulsion stabilized by CNFs with 1.0 mg/mL fucoxanthin showed the highest freeze–thaw, centrifugal, and stored stability. In the Pickering emulsion with 1.0 mg/mL fucoxanthin, the highest encapsulation efficiency was 70.84 ± 3.91%. Moreover, the 1.0 mg/mL fucoxanthin emulsion showed high stability and antioxidant activity under different pH values, salinities, temperatures, and UV irradiation values. Future studies should focus on in vivo validation of release mechanisms and bioavailability. Furthermore, studies should address scalability for industrial production and performance in real food systems to enable commercialization. The research advances sustainable marine delivery platforms for nutraceuticals and fortified foods, bridging the gap between laboratory innovation and practical application.

## Figures and Tables

**Table 1:** The polymerization degree and the crystallinity index (CrI) of CNFs treated by different homogenization pressures.

Sample	Degree of Polymerization	CrI (%)
Microcrystalline cellulose	170.82 ± 4.1 ^c^	74.96 ± 0.74 ^a^
H-0	331.24 ± 3.6 ^a^	65.23 ± 2.96 ^b^
H-35	323.18 ± 15.3 ^a^	51.67 ± 1.14 ^c^
H-70	312.78 ± 10.6 ^a^	65.13 ± 0.96 ^b^
H-105	291.98 ± 11.1 ^ab^	59.82 ± 2.59 ^b^
H-140	264.94 ± 10.5 ^b^	59.23 ± 2.66 ^b^

Different letters (a–c) indicate significant differences between groups (*p* < 0.05).

## Data Availability

The data presented in this study are available on request from the corresponding author.

## Supplementary Materials

The following supporting information can be downloaded at https://www.mdpi.com/article/10.3390/md23080311/s1: Figure S1: The effect of different single factor on the emulsification index in CNFs Pickering emulsion. (A) Oil phase volume fraction;(B) Homogenizing time;(C) CNFs mass fraction;(D) Homogenization speed; Table S1: Factors and levels of orthogonal experiment; Table S2: Orthogonal test results of emulsion preparation Table S3: Analysis of variance of orthogonal test results.
