# Supplementary material for: Enhancing Fucoxanthin Pickering Emulsion Stability and Encapsulation with Seaweed Cellulose Nanofibrils Using High-Pressure Homogenization
Source: Mar Drugs. 2025 Jul 30;23(8):311. doi: 10.3390/md23080311 (PMC12387411; doi:10.3390/md23080311)
Supplement: Supplementary file 1 [file marinedrugs-23-00311-s001.zip › marinedrugs-3778521-supplementary.pdf]

Supplementary File

Enhancing Fucoxanthin Pickering Emulsion Stability and Encapsulation with Seaweed Cellulose Nanofibrils Using High-Pressure Homogenization

Ying Tuo[1], Mingrui Wang[1], Yiwei Yu[1], Yixiao Li[1], Xingyuan Hu[1], Long Wu[1,2,3], Zongpei Zhang[4] , Hui Zhou[1,2,3], Xiang Li[1,2,3]*

[1] College of Food Science and Engineering, Dalian Ocean University, Dalian 116023, China

[2] Dalian Jinshiwan Laboratory, Dalian 116034, China

[3] National R&D Branch Center for Seaweed Processing, Dalian Ocean University, Dalian 116023, China

[4] Qingdao bright moon seaweed group co., LTD, Qingdao 266499, China

* Corresponding author at: College of Food Science and Engineering, Dalian Ocean University, Dalian 116023, China. Tel: +86 411 84763135; Fax: +86 411 84763135; E-mail address: lx910702@163.com

1. Methods

1.1 The effect of different oil phase on emulsion stability

To prepare the emulsion stabilized by the different oil phases, the CNFs (0.5 wt%, pH = 7.0) was mixed with different ratios of the oil phase (φ = 5%, 10%, 15%, 20%, and 25%). Thereafter, the mixture was sheared at 10000 rpm for 120 s using a a homogenizer (AH-BASIC, GEA Niro Soavi, Parma, Italy) to obtain the emulsion. Subsequently, the emulsions were centrifuged at 3000 rpm (25 °C) for 5 min, and the emulsification index was calculated.

1.2 The influence of different homogenization time on emulsion stability

To prepare the emulsion stabilized by the different homogenization time, the CNFs (0.5 wt%, pH = 7.0) was mixed with 10% oil phase volume fraction. Then, the mixture was sheared at 10000 rpm for 30 s, 60 s, 90 s, and 120 s to obtain the emulsion. The emulsions were centrifuged at 3000 rpm (25 °C) for 5 min, and the emulsification index was calculated.

1.3 The influence of different weight fraction of the CNFs on emulsion stability

To prepare the emulsion stabilized by the different weight fractions of the CNFs (pH = 7.0,0.2%, 0.5%, 0.8%, 1.1%, and 1.4%) was mixed with 10% oil phase volume fraction. Then, the mixture was sheared at 10000 rpm for 120 s to obtain the emulsion. The emulsions were centrifuged at 3000 rpm (25 °C) for 5 min, and the emulsification index was calculated.

1.4 The influence of different homogenization speed on emulsion stability

To prepare the emulsion stabilized by the different homogenization speed, the CNFs (0.5 wt%, pH = 7.0) was mixed with 10% oil phase volume fraction. Then, the mixture was sheared at 8000, 10000, 12000, 14000, and 16000 rpm for 120 s to obtain the emulsion. The emulsions were centrifuged at 3000 rpm (25 °C) for 5 min, and the emulsification index was calculated.

1.5 Orthogonal Experiment

As shown in Table S1 the factors and levels of orthogonal experiment .Based on single-factor experiments, the $L_9(3)^4$ orthogonal experiment was conducted with factors A (Nanocellulose weight fraction), B (Homogenization Time), C (Homogenization Speed), and D (Oil Phase Volume Fraction) to optimize the preparation parameters of the CNFs Pickering emulsion.

Table S1. Factors and levels of orthogonal experiment

	**A<br>Nanocellulose weight fraction (wt%)**	**B<br>Homogenization time（s）**	**C<br>Homogenization speed（rpm）**	**D<br>Oil phase volume fraction （%）**
1	0.95	105	9000	8
2	1.1	120	10000	10
3	1.25	135	11000	12

2 Results and Discussion

2.1 Influence of different oil phase on the stability of emulsions

As shown in Figure S1(A), with the increasing oil phase volume fraction, the emulsification index of emulsion initially increases and then decrease. At an oil phase volume fraction of 10% (At 10% oil phase), the emulsification index was 81.53±3.57%. The result was

significantly higher than other oil phase volume fractions (p<0.05). Consequently, an oil phase volume fraction of 10% was chosen for the preparation of subsequent emulsions.

2.1 Influence of different homogenization time on the stability of emulsions

As illustrated in Figure S1(B), with the increasing homogenization time, the emulsification index of emulsion initially increases and then decrease. While the homogenization time was 120 s, the emulsification index was 47.02±1.53%. The result was significantly higher than other homogenization time groups (p<0.05). Consequently, the homogenization time of 120 s was chosen for the preparation of subsequent emulsions.

2.1 Influence of different mass fraction of the CNFs on the stability of emulsions

As shown in Figure S1(A), with the mass fraction of the CNFs increased from 0.2% to 1.1%, the emulsification index of the emulsion was increased. At a mass fraction of 1.1% was significantly higher than the previous three levels(p<0.05). Furthermore, there was no significantly difference in the emulsion with 1.1% (85.32±0.69%) and 1.4% (83.73±1.83%) mass fractions of the CNFs. This result might because the CNFs reached interface saturation at 1.1%, maintaining a constant interfacial tension. Therefore, the mass fraction of 1.1% was selected for the subsequent preparation of emulsions.

2.1 Influence of different homogenization speed on the stability of emulsions

As shown in Figure S1(D), the influence of different homogenization speed on the emulsification index of emulsion was evaluated. The result indicated that the emulsion at 10000 rpm (83.33±1.19%) exhibited a significantly higher emulsification index than those at 14000 rpm (50.00±2.38%) and 16000 rpm (67.86±2.06%) (p<0.05). This suggest that the emulsion system formed at 10000 rpm was more stable. Therefore, a homogenization speed of 10000 rpm was chosen for the subsequent preparation of emulsions.

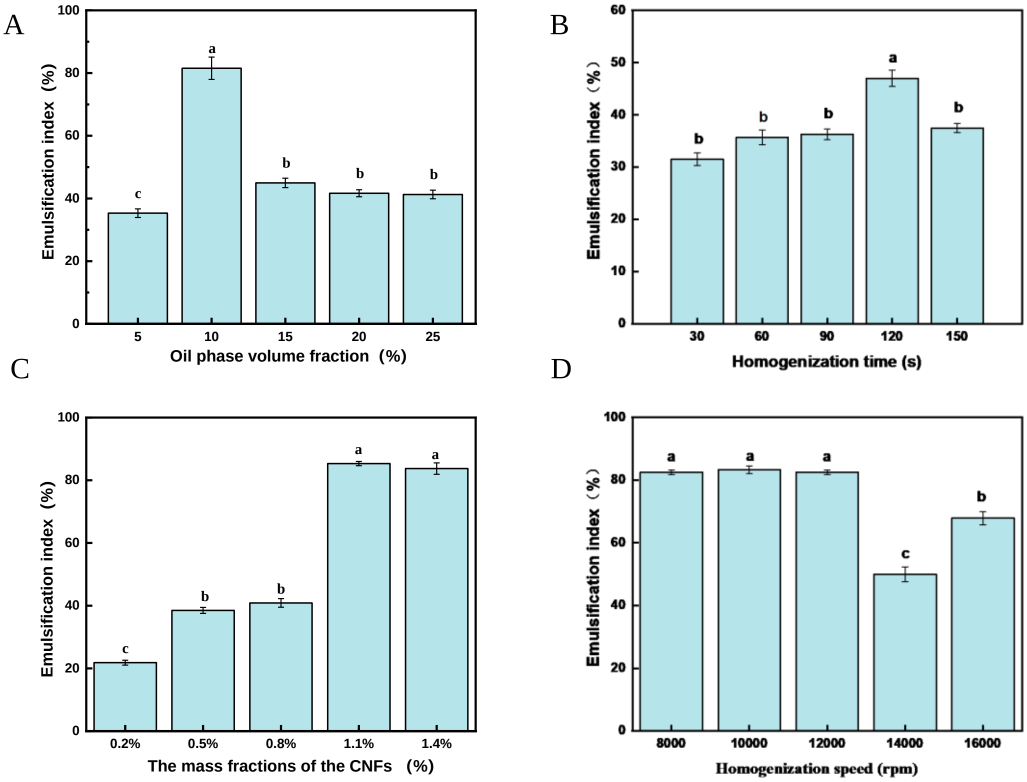

Figure S1. The effect of different single factor on the emulsification index in CNFs Pickering emulsion. （A) Oil phase volume fraction;(B) Homogenizing time;(C) CNFs mass fraction;(D) Homogenization speed. Different letters (a-c) indicate significant differences between groups ($p < 0.05$).

2.2 Outcomes of orthogonal test optimization

After conducting single-factor experiments, appropriate levels of experimentation were selected for oil phase volume fraction, homogenization time, CNFs mass fraction and homogenization speed to perform an $L_9(3^4)$ orthogonal array test. Tables S2 and S3 present the results of the orthogonal experiments for the preparation of emulsion and the subsequent variance analysis of these experiments.

Table S2. Orthogonal test results of emulsion preparation

**Experiment number**	**A The CNFs weight fraction (%)**	**B Homogenizing time (s)**	**C Homogenizing speed (rpm)**	**D Oil phase volume fraction (%)**	**E Emulsification index (%)**
1	0.95	105	9000	8	79.37
2	0.95	120	10000	10	82.94
3	0.95	135	11000	12	48.02
4	1.1	105	10000	12	84.13
5	1.1	120	11000	8	84.92
6	1.1	135	9000	10	86.9
7	1.25	105	11000	10	84.13
8	1.25	120	9000	12	83.73
9	1.25	135	10000	8	86.11
K_1	210.33	247.63	250	250.4	
K_2	255.95	251.59	253.18	253.97	
K_3	253.97	221.03	217.07	215.88	
k_1	70.11	82.54333	83.33333	83.46667	
k_2	85.3166667	83.86333	84.39333	84.65667	
k_3	84.6566667	73.67667	72.35667	71.96	
R	15.2066667	10.18667	12.03667	12.69667	
Optimal Level	A_2	B_2	C_2	D_2	

As shown in Table S2, the four factors influencing the emulsification index were ranked from the most to the least significant as A>D>C>B, with the optimal combination was $A_2B_2C_2D_2$. Since this combination was not included in the orthogonal test table, three additional trials were conducted to verify it, resulting in an emulsification index of 91.43±4.33%. Therefore, the optimal manufacturing parameters for the emulsion were as follow: the oil phase volume fraction of 10%, the homogenization time of 120 s, the CNFs weight fraction of 1.1%, and the homogenization speed of 10000 rpm. As indicated by the P-values in Table S3, factor A (the CNFs weight fraction), B (homogenization time), C (homogenization speed) and D (oil phase

volume fraction) all have a significant impact on the emulsification index of the CNFs Pickering emulsion (P<0.001).

Table S3. Analysis of variance of orthogonal test results

Factor	Type III sum of squares	Degrees of freedom	Mean square	F-value	Significance
A Nanocellulose weight fraction (%)	1330.625	2	665.312	309.146	0.000
B Homogenizing time (s)	552.091	2	276.046	128.268	0.000
C Homogenizing speed (rpm)	799.635	2	399.817	185.780	0.000
D Oil phase volume fraction (%)	885.299	2	442.649	205.683	0.000
Error	38.738	18	2.152		
Total	176520.692	27			

In this study, the optimal conditions were identified as an oil phase volume fraction of 10%, a homogenization time of 120 s, a CNFs weight fraction of 1.1%, and a homogenization speed of 10000 rpm. The orthogonal experiment results showed that the CNFs weight fraction had the most significant impact on emulsification index, followed by oil phase volume fraction, homogenization speed, and homogenization time (P<0.001).

3. Conclusion

In conclusion, this supporting study optimized the preparation of Pickering emulsions through single-factor and orthogonal experiments. The optimal conditions were identified as an oil phase volume fraction of 10%, a homogenization time of 120 s, a CNFs weight fraction of 1.1%, and a homogenization speed of 10000 rpm. The orthogonal experiment results showed that the CNFs weight fraction had the most significant impact on emulsification index, followed by oil phase volume fraction, homogenization speed, and homogenization time (P<0.001). These results provide a scientific basis for stabilizing the of the CNFs Pickering emulsion, which can guide future research and industrial applications to enhance the stability of such compounds in food products.

Enhancing Fucoxanthin Pickering Emulsion Stability and Encapsulation with Seaweed Cellulose Nanofibrils Using High-Pressure Homogenization

Table of Contents

1. Introduction

2. Materials and Methods

2.1. Materials

2.2. Isolation of brown seaweed cellulose

2.3. Preparation of the cellulose nanofibrils (CNFs)

2.4. Determination on the polymerization degree of the CNFs

2.5. X-ray diffraction analysis of the CNFs

2.6. Fourier-Transform infrared spectroscopy (FTIR) analysis of the CNFs

2.7. Determination on particle size and Zeta potential of the CNFs

2.8. Contact angle analysis of the CNFs

2.9. Morphology observation of the CNFs

2.10. Thermogravimetric analysis of the CNFs

2.11. Preparation of the fucoxanthin emulsion stabilized by the CNFs

2.12. Determination on encapsulation efficiency of fucoxanthin in Pickering emulsion

2.13. Analysis on centrifugal stability of the fucoxanthin emulsion

2.14. Analysis on freeze-thaw stability of the fucoxanthin emulsion

2.15. Determination on interfacial adsorption amount of the CNFs

2.16. Preparation of the fucoxanthin emulsion with different pH values

2.17. Preparation of the fucoxanthin emulsion with different salinity

2.18. Preparation of the fucoxanthin emulsion with different temperature

2.19. Preparation of the fucoxanthin emulsion with different UV light exposure duration

2.20. Encapsulation efficiency of fucoxanthin in the Pickering emulsion with different processing and storage factors.

2.21. DPPH free radical scavenging rate of the fucoxanthin in Pickering emulsion

2.22. ABTS cation radical scavenging rate of the fucoxanthin in Pickering emulsion

2.23. Statistical Analysis

3. Results and Discussion

3.1. Effect of homogenization pressure on polymerization degree of brown seaweed cellulose

3.2 Effect of homogenization pressure on crystalline structure of brown seaweed cellulose

3.3. Effect of homogenization pressure on chemical construction of brown seaweed cellulose

3.4. Effect of homogenization pressure on particle size of cellulose nanofibers

3.5. Effect of homogenization pressure on Zeta potential of cellulose nanofibers

3.6. Effect of homogenization pressure on contact angle of cellulose nanofibers

3.7. Effect of homogenization pressure on microstructure of cellulose nanofibers

3.8. Effect of homogenization pressure on thermogravimetric stability of cellulose nanofibers

3.9. Encapsulation efficiency of fucoxanthin in Pickering emulsion

3.10. The stability of the fucoxanthin emulsion

3.11. Interfacial adsorption amount of the cellulose nanofibers in fucoxanthin emulsion

3.12. Effect of pH value on the stability of fucoxanthin emulsion

3.13. Effect of salinity on the stability of fucoxanthin emulsion
3.14. Effect of temperature on the stability of fucoxanthin emulsion
3.15. Effect of UV exposure on the stability of fucoxanthin emulsion
3.16. Effect of storage time on the stability of fucoxanthin emulsion
3.18. Effect of salinity on the antioxidant activity of fucoxanthin in Pickering emulsions
3.19. Effect of temperature on the antioxidant activity of fucoxanthin in Pickering emulsions
3.20. Effect of UV irradiation on the antioxidant activity of fucoxanthin in Pickering emulsions
3.21. Effect of storage time on the antioxidant activity of fucoxanthin in Pickering emulsions
4. Conclusion
5. Author Contributions
6. Declaration of Competing Interest
7. Funding
8. Informed Consent Statement
9. Acknowledgments
10. Supplementary Materials
11. References
